# Enzymes provide demographers with food for thought

**DOI:** 10.7554/eLife.00340

**Published:** 2012-12-13

**Authors:** Mark Jit, Patrick Gerland

**Affiliations:** **Mark Jit** is in the Modelling and Economics Unit, Health Protection Agency, London, UK, and the Department of Infectious Disease Epidemiology, London School of Hygiene and Tropical Medicine, London, United Kingdommark.jit@hpa.org.uk; **Patrick Gerland** is in the Population Division, Department of Economic and Social Affairs, United Nations, New York, United Statesgerland@un.org

**Keywords:** enzyme kinetics, child survival, smoking, income, adult survival, Human

## Abstract

Life expectancy has increased by 20 years since the middle of the last century, but children under five have fared better than adult males.

**Related research article** Hum R, Jha P, McGahan A, Cheng Y-L. 2012. Global divergence in critical income for adult and childhood survival: analyses of mortality using Michaelis–Menten. *eLife* **1**:e00051. doi: 10.7554/eLife.00051

**Image** An equation used to model enzyme kinetics can be adapted to model the link between life expectancy (LE) and GDP

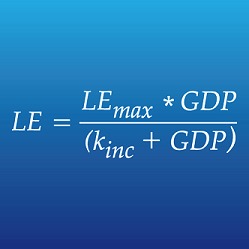

One of the great success stories of the last century is the dramatic increase in the longevity of world citizens. Global life expectancy leaped from 48 years in 1950–1955 to 68 years in 2005–2010 (United Nations, 2011). Major improvements were seen in almost all regions—rich and poor, east and west—with the exception of the former communist bloc countries of Eastern Europe and the countries affected most severely by the HIV/AIDS epidemic in sub-Saharan Africa. Where have these gains come from? Which population groups and which countries have benefitted most? Now, writing in *eLife*, researchers at the University of Toronto explore this question using statistical models that relate life expectancy to national income (Hum et al., 2012). They argue that gains in low-income countries have mainly come from improvements in child survival. Conversely, improvements in adult survival, particularly for males, have been largely restricted to high-income countries. Moreover, the level of national income needed to attain the best possible levels of survival for adults has been steadily rising.

This is not the first statistical model to relate income with life expectancy. In a landmark essay published 37 years ago, the American sociologist Samuel Preston, then at the University of Washington, used a logistic function to model the nonlinear relationship between income and health, and showed that higher national incomes per capita led to longer life expectancies (Preston, 1975). Preston's work has had a lasting influence on research into the relationship between income inequality and health. In 2006, using more recent data, Rati Ram of Illinois State University confirmed what Preston had found, even after controlling for factors such as female illiteracy, secondary school enrolment and ethnic heterogeneity (Ram, 2006). This relationship can also be modelled through the use of smoothing and least-squares fits (Deaton, 2004; Schnabel and Eilers, 2009) without imposing any functional form to fit the data, but in this approach it is not possible to reduce the data to an analytical expression with a few parameters that have clear interpretations. The mathematical formula derived by the Toronto group, on the other hand, provides additional insights into the relationship between national income and life expectancy, and also introduces (through, for example, asymptotic limits) new parameters beyond those that have been studied before.

The Toronto group—Ryan Hum, Prabhat Jha, Anita McGahan and Yu-Ling Cheng—deploy a mathematical relationship called the Michaelis–Menten function, which is more familiar to biochemists than demographers. This function describes the action of an enzyme—a biological catalyst that speeds up a chemical process without getting used up in the process, which means that it can be reused again and again. Hum and colleagues see investments in healthcare that tend to benefit a country over the long term, such as the building of hospitals and the deployment of medical technology, as a kind of enzyme that converts national income into longevity.

So what do they find? First the good news: child survival has dramatically improved. Although this is not the focus of their paper, it is surely an achievement to be celebrated. This reduction in childhood mortality is the fruit of decades of investment in research and the deployment of technologies such as sanitation, maternal services, vaccines, oral rehydration, antibiotics and vitamins. Devastating childhood diseases such as smallpox, polio, measles and beri-beri are now thankfully things of the past in most (though sadly not all) parts of the world. Yet the task is not over—children still die of causes like diarrhoea and pneumonia that can be prevented by administering a vaccine or a simple rehydrating solution. Diarrhoea and pneumonia remain the leading causes of death among children, and it has been estimated that they killed some 2.2 million children under the age of five in 2010, which is almost 30% of the deaths in this age group (Liu et al., 2012).

Still more troubling are the signs that gains in adult survival are becoming more expensive—and that male adult survival in particular has remained flat even though countries have become richer. HIV and smoking-related diseases are singled out by Hum and colleagues as the likely culprits, though a host of other causes may also be to blame: drug resistant tuberculosis, road traffic accidents, air pollution, and alcohol and drug abuse. Unfortunately, unlike many of the causes of death during childhood in low-income countries, these sources of mortality are often exacerbated rather than ameliorated by development. The World Health Organisation (WHO) has estimated that more than 90% of deaths due to these causes occur in low- and middle-income countries, where they represent about 12% of all deaths, compared with 6% for high-income countries (WHO, 2008; WHO, 2009).

Children still die of causes like diarrhoea and pneumonia that can be prevented by administering a vaccine or a simple rehydrating solution.

Hum and colleagues point out that there are cost-effective interventions against lung cancer, HIV/AIDS and many of the other non-communicable diseases that kills adults, such as anti-smoking or safe sex programmes. However, despite being inexpensive, such interventions are often more complex to implement than pharmaceutical interventions such as vaccines and antibiotics. This is because they involve addressing the multiple risk factors that are associated with non-communicable diseases, and because they require a shift from a purely biomedical model of health to one that takes into account the socioeconomic, cultural, legislative and environmental framework in which people live. Achieving this requires collaboration between government departments as diverse as health, agriculture, education, urban planning and infrastructure.

Nevertheless, the work of the Toronto team highlights the necessity of dealing with these causes of mortality if the health gains of the last century are to be maintained and extended into the next. One of the targets set at the sixty-fifth World Health Assembly, held in Geneva in May, was for a 25% reduction in premature mortality from the four major non-communicable killers—cardiovascular disease, cancer, diabetes and chronic respiratory diseases (WHO, 2012). However, for this resolution to be more than aspirational will require a concerted effort on the scale of the campaigns against childhood diseases seen in the last century.
